# The Comparative Study of the Antioxidant and Antibacterial Effects of Propolis Extracts in Veterinary Medicine

**DOI:** 10.3390/vetsci11080375

**Published:** 2024-08-15

**Authors:** Dovile Svetikiene, Gintaras Zamokas, Monika Jokubaite, Mindaugas Marksa, Liudas Ivanauskas, Lina Babickaite, Kristina Ramanauskiene

**Affiliations:** 1 Department of Dr. L. Kriauceliunas Small Animal Clinic, Faculty of Veterinary Medicine, Lithuanian University of Health Sciences, Tilzes Str. 18, LT-47181 Kaunas, Lithuania; gintaras.zamokas@lsmu.lt (G.Z.); lina.babickaite@lsmu.lt (L.B.); 2 Department of Drug Chemistry, Faculty of Pharmacy, Lithuanian University of Health Sciences, Sukileliai Avenue 13, LT-50162 Kaunas, Lithuania; monika.jokubaite@lsmu.lt; 3 Department of Analytical and Toxicological Chemistry, Faculty of Pharmacy, Lithuanian University of Health Sciences, Sukileliai Avenue 13, LT-50162 Kaunas, Lithuania; mindaugas.marksa@lsmu.lt (M.M.); liudas.ivanauskas@lsmu.lt (L.I.); 4 Department of Clinical Pharmacy, Faculty of Pharmacy, Lithuanian University of Health Sciences, Sukileliai Avenue 13, LT-50162 Kaunas, Lithuania

**Keywords:** animal, antimicrobial, propolis, extract

## Abstract

**Simple Summary:**

Bacterial congenital diseases are among the most common ailments in dogs and cats within veterinary medicine. Microorganisms are becoming more and more resistant to antibiotic drugs. The search for natural substances has been driven primarily by the irrational and reckless use of available antimicrobials in clinical scenarios. Consequently, there is a growing interest in natural substances for treating animal diseases, aiming to find sources of active compounds of natural origin. Propolis is one such substance that scientists have extensively investigated. It has garnered contemporary interest due to its natural complex of active compounds and broad biological activity. This study demonstrates propolis extracts’ chemical analysis and biological activity using different solvents. The bee propolis extracts’ antimicrobial and antifungal activities were evaluated using clinical and reference bacterial strains.

**Abstract:**

Antimicrobial resistance (AMR) is one of the biggest threats to human and animal health. Efforts to combat AMR include the introduction of antimicrobial drugs as alternative treatment options. To contribute to an effective plan for the treatment of infectious diseases caused by bacteria, the development of new antimicrobial agents is increasingly being explored. Propolis has garnered significant attention from both scientists and industry due to its extensive spectrum of biological activity. The growing interest in polyphenols of natural origin and their plant sources further encourages the investigation of their chemical composition and biological effects. Propolis serves as a rich source of phenolic compounds. Baltic region propolis, classified as poplar-type propolis, was selected for this study, and extracts were prepared using raw propolis materials from various Baltic countries. The production of liquid extracts utilized a combination of 70 percent ethanol, a mixture of water and poloxamer P407, and DES (deep eutectic solvent). The research aims to produce liquid propolis extracts using different solvents and to assess their chemical composition, antioxidant, and antimicrobial activity against different veterinary pathogens. Antioxidant activity was evaluated using DPPH (2,2-diphenyl-1-picrylhydrazyl), revealing antioxidant activity in all extracts, with results correlating with the total phenolic compound content. It was found that *p*-coumaric acid predominated in the studied propolis extracts (in ethanol extracts 1155.90–1506.65 mg/g, in DES extracts 321.13–954.76 mg/g, and in polymeric extracts 5.34–30.80 mg/g), with smaller amounts of ferulic acid and vanillin detected. Clinical and reference bacterial strains were collected from the Lithuanian University of Health Sciences, the Academy of Veterinary Medicine, and the Institute of Microbiology and Virology. To effectively treat bacterial infections, the antimicrobial activity of propolis extracts was tested against six pathogenic bacterial species and one pathogenic fungus (*S. aureus*, *S. agalactiae*, *B. cereus*, *E. faecalis*, *E. coli*, *P. aeruginosa*, and *C. albicans*). Antimicrobial activity studies demonstrated that DES propolis extracts exhibited stronger antimicrobial activity compared to ethanolic propolis extracts. The minimum inhibitory concentration (MIC) values of DES propolis extracts against the tested strains ranged between 50 and 1000 μg/mL. Considering the study results, it can be concluded that propolis from the Baltic region is abundant in phenolic compounds exhibiting antioxidant and antibacterial activities.

## 1. Introduction

Natural products are important as medicines in many treatment areas [1]. With the discovery of antimicrobial agents almost a century ago, several classes of antimicrobial agents of natural origin, such as β-lactams, tetracyclines, and aminoglycosides, have been introduced as therapeutic agents. At that time, the use of antimicrobials had a significant impact on the treatment of infectious diseases in human and veterinary medicine. Domestic animals are considered potential carriers of antimicrobial resistance (AMR) to humans due to the excessive use of antimicrobials and their close contact with humans. The World Health Organization (WHO) considers antimicrobial resistance to be a major threat to human and animal health. These developments are strongly influenced by the misuse or overuse of antimicrobials, leading to the spread of bacterial antimicrobial resistance (AMR) [2,3,4]. There is now a worldwide increase in the use of plant-based products. Production animals account for around 70% of animals treated with herbal products, poultry (9.1%), dogs (5.3%), and rabbits (4.3%) [5]. One of the threats of antimicrobial resistance (AMR) is the presence of antibiotic residues, which promotes the development of antibiotic-resistant bacteria in humans; toxic metabolites remain in the meat, and the by-products of synthetic substances become a concerning side effect of medication use. These aspects encourage the search for safe alternatives compared to modern animal health systems [6]. The search for natural and safe alternatives to traditional veterinary medicines has become an important area of research in animal and veterinary medicine. This includes natural products or components extracted from plants, microorganisms, insects, or marine organisms. As natural products from different sources have unique structures and properties and do not cause significant side effects, their effect is hoped to reduce the likelihood of drug resistance [7]. The plant kingdom is currently garnering significant attention. Plants are characterized by compounds with a strong antibacterial effect, which are effective in treating bacterial infections without causing adverse side effects. Natural products have a chemical diversity of bioactive compounds so that they can be promising sources for drug development [8,9,10]. Bacterial skin, eyes, and ear infections are among the most common bacterial infections in dogs and cats in veterinary medicine. Anti-inflammatory and antibacterial agents are commonly included in the treatment regimens for many inflammatory diseases. With the growing popularity of natural substances among pet owners, there is an increasing demand for natural veterinary preparations. In this study, it was decided to focus on propolis, one of the substances extensively studied by scientists. Propolis is a bee product known for its natural complex of active compounds and its broad spectrum of biological effects.

Bees collect propolis from living plants, which contributes to the diversity of its chemical composition and, therefore, presents challenges in standardizing its quality. Between 80 and 100 chemical compounds have been identified in propolis samples, most of which are classified as phenolic compounds. These phenolic compounds account for the wide range of biological effects of propolis and, due to their potential health benefits, have garnered significant attention in the scientific community [11]. Recently, scientists have been focusing extensively on polyphenolic compounds as antioxidants. According to epidemiological studies and related meta-analyses, long-term consumption of phenolic compounds is associated with protection against cancer, cardiovascular diseases, diabetes, osteoporosis, and neurological disorders [12,13]. Scientific research results confirm propolis’s antioxidant activity [14,15,16,17,18,19,20,21,22,23,24]. Currently, researchers are working to determine the relationship that the antioxidant activity and individual chemical composition of propolis have. The results of studies on antioxidant activity conducted by Polish scientists have confirmed that the composition of propolis varies depending on the geographical region of collection and plant sources [25,26,27]. Furthermore, these studies demonstrated a dependence of antioxidant activity on the chemical composition of the samples [28]. Shigenori Kumazawa et al. found that ethanolic propolis extracts from some countries (e.g., Argentina, China, Hungary) exhibited strong antioxidant activity, correlated with polyphenol and flavonoid content [29]. Propolis is widely used in cosmetic, pharmaceutical, veterinary, and medical products due to its antibacterial properties. The antibacterial effects of propolis are examined in different ways. First, the antimicrobial activity directly impacts the microorganism, and second, it boosts the immune system by activating the body’s defense mechanisms [30,31]. The antimicrobial activity of propolis is stronger against Gram-positive bacteria than against Gram-negative bacteria [30,32,33,34]. Ethanol predominantly produces propolis extracts, with aqueous extracts being less common. Propolis has a lower water solubility than ethanol solvents. Solvents such as PEG, propylene glycol, and cyclodextrins have been used in aqueous formulations to improve the solubility of these compounds. Korean researchers utilized poloxamers as solubilizers and gelling agents in topical antimicrobial formulations of propolis [35]. Poloxamers can improve the solubility of active compounds by reducing surface tension and forming micelles, which promote the dissolution of active substances [36]. Deep eutectic solvents (DES) have been increasingly used to produce extracts. The results of antimicrobial studies by Trusheva and other researchers have confirmed that DES can improve the antimicrobial effect of propolis extracts on pathogenic microorganisms [37]. This may be influenced by the presence of natural acids as DES elements. The literature indicates that DES containing organic acids have a higher antimicrobial activity due to their pharmacological properties [38]. However, research data on propolis extraction using deep eutectic solvents have been limited. Turkish researchers found that natural deep eutectic solvents (NADES) can be a viable alternative to conventional solvents for propolis extraction [39]. Funari’s results strongly suggest that some NADES extracts can replace hydroethanol, propylene glycol, and aqueous propolis solutions without sacrificing extraction efficiency [40]. Considering that the propolis of the Baltic region belongs to the poplar-type propolis, this study chose to make extracts using raw materials from Lithuanian, Polish, and Latvian propolis. For the production of liquid extracts, 70 percent ethanol, a mixture of water and poloxamer P407, and a deep eutectic solvent (DES) were used. The purpose of this research is to produce liquid extracts of propolis using various solvents and evaluate their chemical composition and antioxidant and antimicrobial activity against different veterinary pathogens.

## 2. Materials and Methods

### 2.1. Propolis Extraction

Ethanolic, polymeric, and DES propolis extracts were produced. Unprocessed propolis samples were commercially purchased from various Baltic region countries (Lithuania, Latvia, Poland). Lithuanian propolis was purchased from R. Serksnienes farm, Raseiniu district, Latvian propolis was purchased from a company (“Bites”, Vālodzes, Stopiņu province, Latvia, LV-2130), Polish propolis was purchased from a beekeeping company (PROKIT, Halinów, Poland).

### 2.2. Ethanolic Propolis Extracts Preparation

Crushed propolis was extracted with 70% ethanol (*v*/*v*) by maceration method in a ratio of 1:10. The macerated content was stored in dark glass bottles at 21 ± 1 °C for 12 days, with the contents being stirred several times during this period [24].

### 2.3. DES Propolis Extracts Preparation

Deep Eutectic solvent (DES) was prepared separately, pouring in the same quantity of materials as specified earlier and mixing at a certain temperature. Choline chloride (Sigma-Aldrich, St. Louis, MO, USA), lactic acid (Sigma-Aldrich, St. Louis, MO, USA) and purified water were mixed in a ratio of 1:1:1. Crushed propolis were extracted with DES solvent by maceration method in a ratio of 1:10. The macerated content was stored in dark glass bottles (21 ± 1 °C) for 12 days, while the content being stirred several times [41].

### 2.4. Polymeric Propolis Extracts Preparation

Polymeric solvent was prepared with poloxomer 407 (Fagron, St. Paul, MN, USA). The mixture was stored in the refrigerator (4 ± 1 °C) for 24 h until the polymer completely dissolved. Crushed propolis was extracted by the polymeric solvent in a ratio of 1:10 by maceration. The stirring macerate content was stored in dark glass bottles (4 ± 1 °C) for 12 days and stirred several times in the meantime [35].

The extracts were filtered through ashless filter paper (retention 8–12 µm, diameter 90 mm, ash content 0.007%) [42].

### 2.5. HPLC Analysis

HPLC analysis was performed based on certain modifications [43]. Chromatographic equipment used included a Waters 2695 system with a Waters 996 diode array detector and an ACE 5C18 chromatography column with dimensions of 250 × 4.6 mm. The obtained data were processed using Empower 2 Chromatography Data Software. HPLC eluent: acetonitrile and trifluoroacetate acid; temperature: 25 °C; duration time: 81 min; and sample injection volume: 10 µL. The setting for the analyzed compounds was established by comparing them with benchmark materials, considering their retention time and UV absorption within the range of 250 to 400 nm. Standard compounds used in the analysis: *p*-coumaric acid (R^2^ = 0.9999), cinnamic acid (R^2^ = 0.9999), caffeic acid (R^2^ = 0.9999), vanillin (R^2^ = 0.9999), vanillic acid (R^2^ = 0.9999), and ferulic acid (R^2^ = 0.9999).

### 2.6. Antioxidant Activity in DPPH

The antioxidant activity of the extracts was evaluated using the DPPH method, with some modifications according to Yim et al. [44]. A 60 µM DPPH solution in 96% ethanol (*v*/*v*) was prepared. We mixed 10 µL of propolis extracts with 3000 µL of DPPH working solution. The samples were incubated for 30 min at room temperature. Absorbance was measured with a spectrophotometer (Agilent Technologies 8453 UV-Vis, Santa Clara, CA, USA) at the wavelength of 517 nm.

### 2.7. Antimicrobial Activity In Vitro

Assessment of antimicrobial activity in vitro was determined based on previous studies [32,45]. The bacteriological properties of propolis extracts were evaluated in vitro using the agar diffusion method. Mueller–Hinton agar (Biolife Mueller Hinton Agar II, Italy), Columbia blood agar (CBA, EO Labs, Bonnybridge, Scotland, UK), and Sabouraud dextrose agar (SDA, EO Labs, Bonnybridge, Scotland, UK), approved by the Clinical and Laboratory Standards Institute (CLSI), were used as standards. Liquid Mueller–Hinton and Sabouraud dextrose agars were prepared according to the standards and poured into Petri dishes with a diameter of 10 cm, approximately 35 mL each. Ready-made Colombia blood agar was used. Each isolate was prepared according to the 0.5 McFarland standard. Clinical and reference bacterial strains were collected from the Lithuanian University of Health Sciences, the Academy of Veterinary Medicine, and the Institute of Microbiology and Virology. Small animals with acute superficial or deep pyoderma, wound infections, abscesses, otitis, conjunctivitis, or complicated corneal ulcers were treated. Clinical and reference bacterial strains, including *Staphylococcus aureus* (ATCC 25923), *Bacillus cereus* (ATCC 11778), *Enterococcus faecalis* (ATCC 29212), *Escherichia coli* (ATCC 25922), and *Pseudomonas aeruginosa* (ATCC 27853), were spread onto Columbia blood agar. Additionally, the *Streptococcus agalactiae* (ATCC 13813) strain was disseminated on Columbia blood agar. Clinical and referential *Candida albicans* (ATCC 10231) strains were spread onto Sabouraud dextrose agar. Wells with a diameter of 7 mm were made in the agar, into which 0.1 mL of the propolis extract research material was added. Plates with bacteria were incubated for 24 h at 37 ± 0.5 °C, and plates with fungi were incubated for 24 h at 35 ± 0.5 °C in the thermostat. The antibacterial and antifungal properties of the propolis extracts in vitro were assessed after 24 h of incubation. The diameter of sterile zones formed around the wells was calculated in millimeters.

### 2.8. Minimum Inhibitory Concentration

The minimum inhibitory concentration (MIC) was determined using the broth microdilution method as recommended by the Clinical and Laboratory Standards Institute. The MIC was defined as the lowest concentration of the propolis extract that inhibited the growth of the tested microorganisms. The test was performed on clinical and reference bacterial strains, including *Staphylococcus aureus* (ATCC 25923), *Streptococcus agalactiae* (ATCC 13813), *Bacillus cereus* (ATCC 11778), *Enterococcus faecalis* (ATCC 29212), *Escherichia coli* (ATCC 25922), and *Pseudomonas aeruginosa* (ATCC 27). For the study, the bacterial strains were initially inoculated in sterile saline (0.9%). Turbidity was adjusted according to the McFarland 0.5 turbidity standard (Densi-La-Meter II, Erba Lachema, Brno, Czech Republic). A 10 µL aliquot of the diluted bacterial suspension was added to 11 mL of Mueller–Hinton Broth II (MHB, Oxoid Ltd., Basingstone, Hampshire, UK) liquid medium, resulting in a final inoculum volume of 1.5 × 10^8^ CFU/mL. The prepared inoculum was then inoculated into 96-well microplates (VWR International, LLC., Radnor, PA, USA). Fifty microliters of medium and 50 µL of various concentrations of the propolis extract were added. The concentrations of propolis extracts tested ranged from 0.05 to 100 mg/mL. The plates were incubated at 37 °C for 24 h. Controls included a negative control (medium only), positive control (medium with bacteria), and color control (extract dilutions). The MIC of *p*-coumaric acid, the predominant compound in the extracts as determined by HPLC analysis, was also estimated. The concentrations of *p*-coumaric acid tested ranged from 0.1 mg/mL to 1 mg/mL. Bacterial growth was evaluated with a Multiskan FC microplate photometer (Thermo Scientific, Foster City, CA, USA) after 24 h of incubation by measuring the absorbance at 570 nm (OD570). Based on the control samples, thresholds were established to classify the results as either bacterial growth or no growth.

### 2.9. Statistical Analysis

The results express averages of three measurements and standard deviations. Statistically significant differences between the compared data were determined using one-way ANOVA. Differences were considered statistically significant when *p* < 0.05. The correlation was evaluated based on the Spearman correlation coefficient. Data statistical analysis and visualization were performed using software tools, including OriginPro^®^ 2021 (OriginLab, Northampton, MA, USA), IBM SPSS Statistics 27 (SPSS Inc., Chicago, IL, USA), and SigmaPlot 13.0 (Systat Software, San Jose, CA, USA).

## 3. Results

### 3.1. HPLC Analysis of Active Compounds

The chemical composition of the produced extracts was evaluated using HPLC (Table 1). The research data indicate that *p*-coumaric acid was predominant in the studied ethanolic extracts, accounting for 19.73% of the total amount of all determined active compounds. Compared to other active substances found in the tested extracts, higher amounts of ferulic acid (9%) and vanillin (7.68%) of the total active compounds were found. Small amounts of cinnamic acid (0.5%) and vanillic acid (0.38%) were also detected. In eutectic extracts, *p*-coumaric acid continued to dominate, comprising about 33% of the total active compounds. Vanillin and ferulic acid accounted for 14% and 10.9%, respectively. Vanillic acid and cinnamic acid were the least found, accounting for 0.67% and 0.03%, respectively, of all identified compounds in the eutectic extracts. Further data show that in polymeric extracts of propolis, smaller amounts of *p*-coumaric acid (31.32%), vanillin (14.60%), and ferulic acid (11.11%) were found. Compared to other substances, the least amounts of vanillic and cinnamic acids were found, making up a minimal percentage of all active compounds.

### 3.2. Antioxidant Activity In Vitro

**Figure 1:**
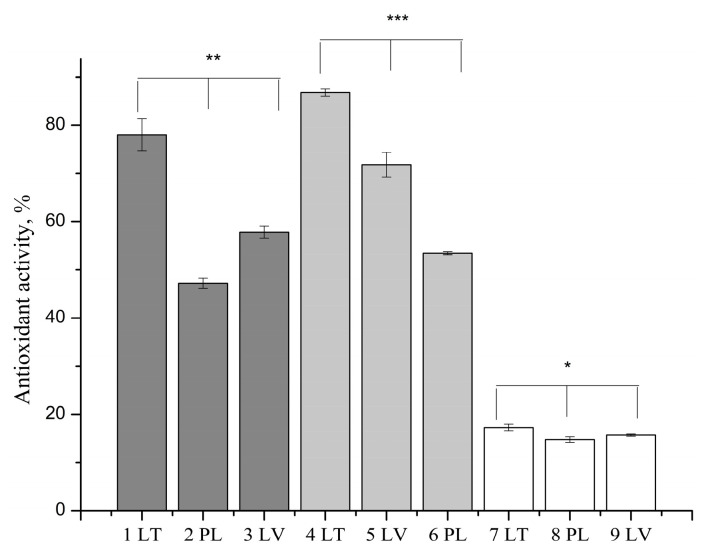
Antioxidant activity of ethanolic, eutectic, and polymeric (P407) propolis extracts. 1 LT, 2 PL, 3 LV—ethanolic extracts, 4 LT, 5 LV, 6 PL—eutectic propolis extracts, 7 LT, 8 PL, 9 LV—polymeric (P407) propolis extracts. Ethanolic propolis extracts were diluted five times before the test. The asterisks indicate statistical significance between polymeric (P407) propolis extracts group compared to other propolis extracts groups, *p* < 0.05.

Antioxidant activity of prepared experimental extracts was analyzed by DPPH method. Research results are presented in Figure 1.

The data presented in Figure 1 show that all tested extracts exhibit antioxidant activity. The ethanolic extract of Lithuanian propolis showed similar antioxidant activity to the propolis DES extract. Using the DPPH method, polymeric (P407) propolis extracts (7 LT, 8 PL, 9 LV) exhibited statistically significantly weaker antioxidant activity compared to other analyzed propolis extracts (*p* < 0.05). The results of the study indicate a strong correlation between the total sum of active phenolic compounds identified through HPLC analysis and the antioxidant activity results (*p* < 0.001).

### 3.3. Determination of Antimicrobial Activity

**Figure 2:**
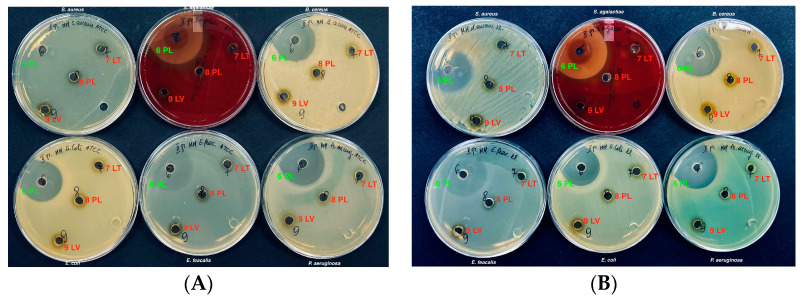
Antimicrobial activity of propolis polymeric (P407) extracts. Reference strains (**A**). Wild strains (**B**).

The antimicrobial activity of ethanolic, eutectic, and polymeric extracts of P407 propolis was studied. Research data in Figure 2, show that propolis polymeric extracts did not exhibit inhibitory antibacterial activity against any tested bacterial strains; bacterial growth was observed around the cavities (7 LT, 8 PL, 9 LV).

**Figure 3:**
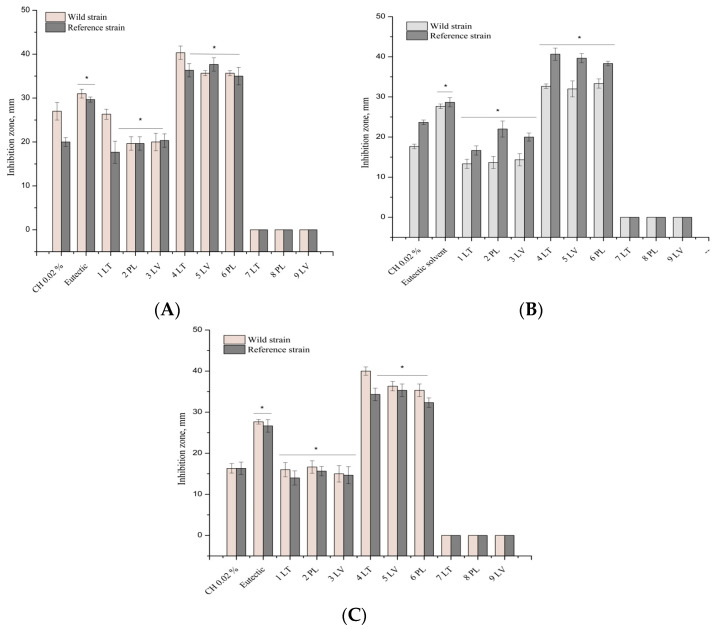
Antibacterial activity of propolis extract on *S. aureus* (**A**), *S. agalactiae* (**B**), and *B. cereus* (**C**) In the figure, an asterisk (*) indicates statistically significant results (*p* < 0.05).

The ethanolic and DES propolis extracts in Figure 3A exhibit antibacterial activity against the reference and clinical bacteria of the *Staphylococcaceae* family. The eutectic propolis extracts were characterized by a stronger inhibitory antibacterial effect compared to the ethanolic propolis extracts. A significant difference (*p* < 0.05) was found between the inhibition zones of these propolis extracts. The tested propolis extracts showed a better antibacterial effect against the reference bacterium of the *Staphylococcaceae* family compared to the clinical strain of *S. aureus*. A statistically significant difference (*p* < 0.05) was found between the inhibition zones of the reference and clinical *S. aureus*. The DES extracts showed a statistically significantly stronger antibacterial inhibitory effect against *S. aureus* compared to the control eutectic solvent (*p* < 0.05) and the positive control. Ethanol propolis extracts exhibited statistically weaker activity compared to the chlorhexidine solution. The ethanolic and DES propolis extracts in Figure 3B demonstrate antibacterial activity against the reference and clinical bacteria of the *Streptococcaceae* family. The DES propolis extracts exhibited a stronger inhibitory antibacterial effect compared to the ethanolic propolis extracts. A significant difference (*p* < 0.05) was observed between the inhibition zones of these propolis extracts. Furthermore, the eutectic propolis extracts were characterized by a statistically significantly stronger antibacterial inhibitory effect against *S. agalactiae* compared to the control eutectic solvent (*p* < 0.05). The DES extracts inhibited the growth of *S. agalactiae* statistically significantly compared to the chlorhexidine control group (*p* < 0.05). The ethanol propolis extracts and chlorhexidine solution exhibited antibacterial activity against the *B. cereus* strain. The ethanol extracts demonstrated a statistically significantly weaker antibacterial effect against bacteria of the *Bacillaceae* family compared to the propolis DES extracts and DES solvents (*p* < 0.05). The inhibition zones of the strains were statistically significantly larger in diameter for the eutectic extracts compared to the ethanol propolis extracts Figure 3C.

**Figure 4:**
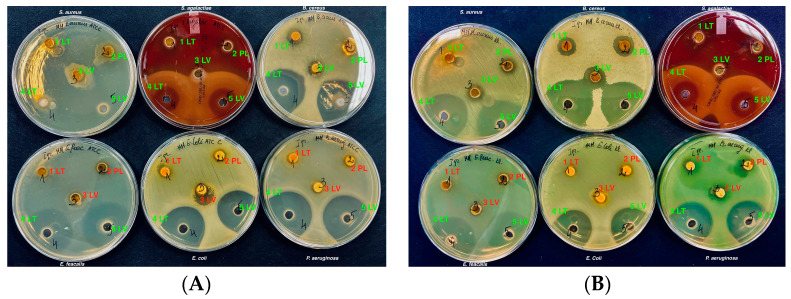
Antimicrobial activity of propolis ethanolic extracts. Reference strains (**A**). Wild strains (**B**). 1—LT ethanolic propolis, 2—PL ethanolic propolis, 3—LV ethanolic propolis, 4—LT eutectic propolis, 5—LV eutectic propolis, 6—PL eutectic propolis, 7—LT polymeric propolis, 8—PL polymeric propolis, 9—LV polymeric propolis.

This study’s results, depicted in Figure 4, showed that the ethanol propolis extracts did not inhibit bacteria from the *Enterococcaceae*, *Enterobacteriaceae*, and *Pseudomonadaceae* families. Bacterial growth was observed around the cavities (1 LT, 2 PL, 3 LV).

**Figure 5:**
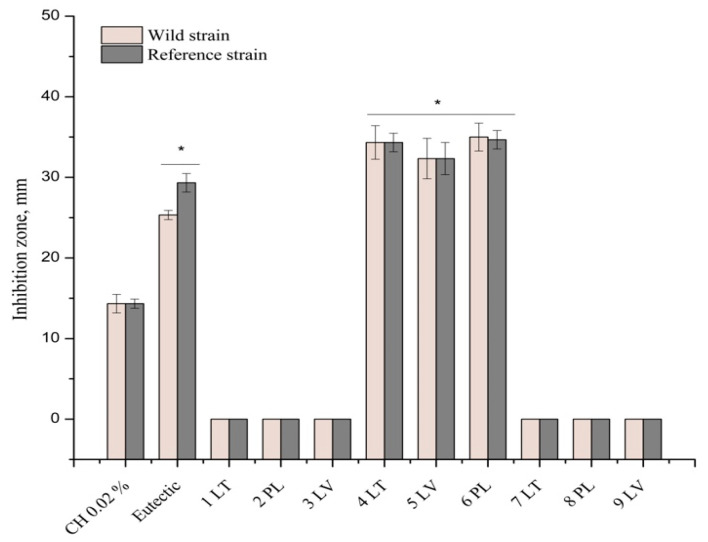
Antibacterial activity of propolis extract on *E. feacalis*. In the figure, an asterisk (*) indicates statistically significant results (*p* < 0.05).

Figure 5 illustrates that the DES propolis extracts exhibited antibacterial activity against reference and clinical *E. feacalis* bacteria of the *Enterococcaceae* family. Statistically significant strain inhibition zones were larger in diameter in the DES extracts compared to the DES solvent and positive control (*p <* 0.05).

**Figure 6:**
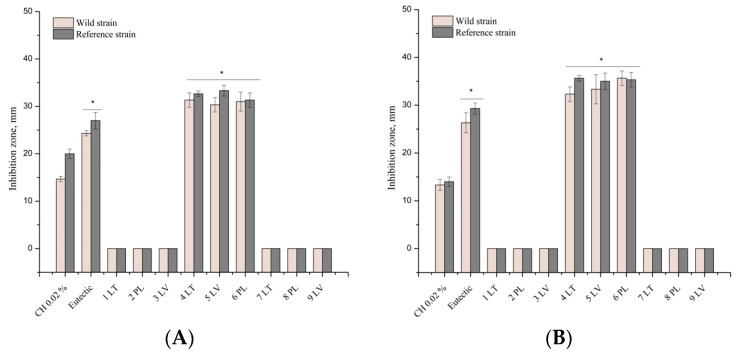
Antibacterial activity of the propolis extract on *E. coli* (**A**), *P. aeruginosa* (**B**). In the figure, an asterisk (*) indicates results that are statistically significant (*p* < 0.05).

During the research, it was found that the DES propolis extracts have strong antimicrobial activity against the Gram-negative bacteria *E. coli* strain. As depicted in Figure 6A, the eutectic propolis extracts exhibit antibacterial activity against the reference and clinical bacteria of the *Enterobacteriaceae* family. Statistically significantly, the zones of inhibition of the tested strains had a wider diameter in the DES extracts compared to the DES solvent and the positive control (*p <* 0.05). During the research, it was found that the DES propolis extracts have excellent antimicrobial activity against the Gram-negative *P. aeruginosa* bacterial strain (Figure 6B). The growth zone of the reference *P. aeruginosa* strain was statistically significantly inhibited by the DES propolis extracts compared to the control DES solvent. The growth of clinical *Pseudomonadaceae* bacteria was statistically significantly inhibited by the DES propolis extracts compared to the chlorhexidine control group (*p <* 0.05).

**Figure 7:**
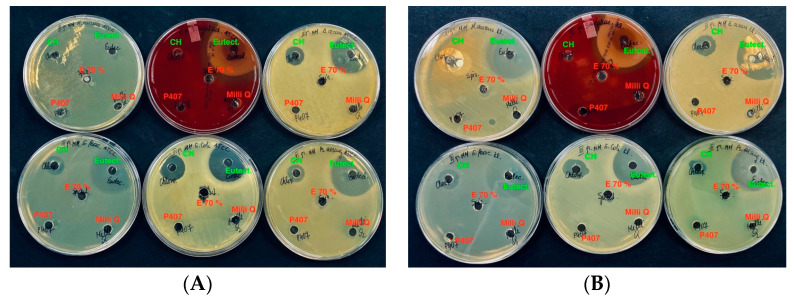
Antimicrobial activity of control groups. Reference strains (**A**). Wild strains (**B**). CH—chlorhexidine aqueous solution 0.02%, Eutect.—eutectic aqueous solution, P407—poloxomer (P407) aqueous solution, E-70%—ethanol, Milli Q.

For the bacterial spot studies, Figure 7 was selected as positive controls, consisting of chlorhexidine 0.02% aqueous solution for the control group. From the results of the study, it was observed that the aqueous solution of chlorhexidine exhibited antibacterial effects. It demonstrated antibacterial activity against both Gram-positive and Gram-negative clinical and reference bacterial strains. The selected eutectic solvent served as a negative control and also showed antibacterial activity against the tested clinical and reference bacterial isolates. The components of the DES solvent contributed significantly to the antibacterial activity of the propolis extracts against the tested bacterial isolates.

**Figure 8:**
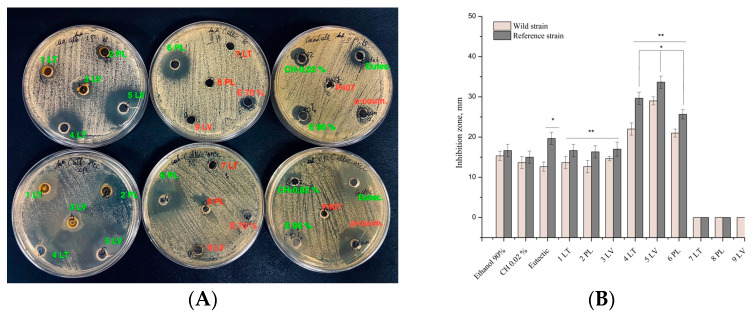
Antifungal activity of the propolis extracts. Referential and wild *C. albicans.* 1—LT ethanolic propolis, 2—PL ethanolic propolis, 3—LV ethanolic propolis, 4—LT eutectic propolis, 5—LV eutectic propolis, 6—PL eutectic propolis, 7—LT polymeric propolis, 8—PL polymeric propolis, 9—LV polymeric propolis, CH—chlorhexidine aqueous solution 0.02%, Eutec.—eutectic aqueous solution, P407—poloxomer (P407) aqueous solution E—90/70%—ethanol (**A**). Antifungal activity of propolis extract on *C. albicans* (**B**). Asterisks indicate statistical significance between the eutectic control and propolis extract groups, *p* < 0.05.

The research data depicted in Figure 8A show that propolis polymeric extracts did not exhibit inhibitory antifungal activity against the clinical and reference fungi of the *Saccharomycetaceae* family. Fungal growth was observed around the cavities (7 LT, 8 PL, 9 LV).

The DES propolis extracts in Figure 8B demonstrated statistically significant antifungal activity against the reference and clinical fungi of the *Saccharomycetaceae* family (*p <* 0.05). The DES propolis extracts exhibited a stronger inhibitory antifungal effect compared to the ethanolic propolis extracts. A significant difference (*p <* 0.05) was found between the inhibition zones of these propolis extracts. Furthermore, the DES propolis extracts showed a statistically stronger antifungal inhibitory effect against *C. albicans* compared to the control solutions (*p <* 0.05).

### 3.4. Minimum Inhibitory Concentration

The MIC results of the present study demonstrated that the antimicrobial activity of the tested propolis extracts ranged from 0.05 mg/mL to 5 mg/mL. The minimum inhibitory concentration of *p*-coumaric acid, the most abundant active compound in the tested extracts, ranged from 0.1 mg/mL to 1 mg/mL (Table 2). Comparison of the values in this study with a previous agar diffusion study clearly shows that the propolis extracts exhibit significant antimicrobial properties against the *Staphylococcus aureus*, *Streptococcus agalactiae*, *Bacillus cereus*, *Enterococcus faecalis*, *Escherichia coli*, and *Pseudomonas aeruginosa* clinical and reference strains.

## 4. Discussion

The emergence and spread of AMR are putting global health systems at risk; for example, the 2015 WHO Global Action Plan on AMR has become one of the key roadmaps in the fight against the elements of AMR, which includes the screening of compounds in order to capture new antimicrobials [46]. Advances in many different research areas have fueled the resurgence of natural products. All of this leads to curbing AMR and better understanding [47]. Veterinary medicine focuses more on herbal medicines to curb the spread of AMR. There is a widespread popular belief that medicinal plants are effective and safer than synthetic compounds. The other main reason is economical, as they are cheaper than conventional therapies and useful in treating subclinical or chronic diseases without conventional treatment, as well as disorders that do not require professional diagnosis [48,49]. The selection of natural products must cover the widest and most diverse range of target pathogens/species (e.g., Gram-positive, Gram-negative bacterial species, spore-forming and acid-fast species, extracellular micro-organisms, clinical isolates) to be able to evaluate the potential of the active compounds [50]. The use of natural materials in veterinary medicine has been the subject of a number of studies on the health and treatment of animals. Several veterinary medicine researchers have investigated the antimicrobial activity of natural products against canine Staphylococcus and *Enterobacteriaceae* spp. These studies investigated various natural sources, including herbal extracts, honey products, and bacteriophages [51,52]. Such studies have provided valuable insights into the potential effectiveness of natural compounds as alternative treatments. Organic sulphur derivatives of garlic, such as allicin compounds, have been reported to have antibacterial activity against *Staphylococcus* spp. [53]. Essential oils are another perspective that is being widely explored. Scientists have studied the activity of oregano essential oil (OEO) and found that it has unique antibacterial and antioxidant properties. These properties have brought OEO to the attention of the whole world so that it can replace antimicrobial growth promoters, which have a significant impact on the livestock industry. OEO has been recommended for the treatment of infections caused by *Candida* spp. [54]. Siddique and other researchers have carried out in vitro probiotic studies with lactic acid bacteria as potential probiotic candidates. Probiotics are beneficial bacteria that live in the gut and help improve health; they are also one of the most popular alternatives to antibiotics [55]. Regarding small animal health disorders that can be treated with medicinal plants or their active compounds, a study was carried out in Spain on the number of animals treated with medicinal plants in clinic visits. The results showed that half of the patients treated in the clinic were treated for dermatological diseases, 70.1% were treated for musculoskeletal diseases, and about 51% of the dogs were treated for supraintestinal disorders [56]. Among the natural plant-based treatment options for veterinary diseases is propolis. For example, propolis has been used as an ointment to control mastitis in cows, as a prophylactic in pig herds for diseases of the respiratory tract, as a gastrointestinal tract, as a growth stimulant, and even as a local anaesthetic during surgery [57]. Propolis has also been used to effectively treat eye diseases in humans and animals due to its many healing properties. In cats and dogs suffering from blepharitis, infectious conjunctivitis, corneal edema, lacrimal duct obstruction, dry keratoconjunctivitis, corneal ulcers, and glaucoma, treatment was administered [58]. Propolis is a natural source of phenolic compounds, and choosing the right solvent for its extraction is important [59]. One of the objectives of this study was to produce propolis extracts with different solvents. For the production of extracts, the maceration method was chosen as the most accessible and most widely used extraction method in practice [60]. Our research results confirm data from the scientific literature that the qualitative profile of phenolic compounds and their quantity in extracts depend on the solvent used in the production and the propolis raw material for extraction. DES components can be selected not only to tune the physicochemical properties of the selected solvents but also to enhance the biological activity of the DES extracts [61,62]. The smallest quantities of the studied active compounds were found in extracts produced using water and a mixture of poloxamer (P407). The application of eutectic solvents allowed for the separation of larger quantities of active compounds; however, these solvents demonstrated weaker extraction capabilities compared to 70% (*v*/*v*) ethanol. Additionally, the application of DES propolis raw material for extraction has paved the way for potential non-ethanol liquid extracts as a basis for adaptation [63]. These extracts showed a statistically significant increase in the total quantity of active compounds compared to extracts produced with poloxamer (P407). Propolis extracts were derived from raw propolis collected in various countries across the Baltic region. The profile of active compounds in the investigated propolis extracts exhibited similarity. The scientific literature indicates that the propolis of Lithuania, Poland, and Latvia is categorized as poplar-type propolis [64,65,66,67]. The highly dominant compound in propolis extracts, *p*-coumaric acid, is recognized as a hydroxyl compound of cinnamic acid. It is known for its ability to reduce the peroxidation of low-density lipoproteins, exhibit antimicrobial activity, contribute to inhibiting cellular melanogenesis, and positively affect the regulation of the human immune system [68]. Lower amounts of ferulic acid and vanillin were found in the tested extracts compared to *p*-coumaric acid. Ferulic acid in propolis extracts is an important natural antioxidant. There is a wealth of published scientific research data on the antioxidant, antiallergic, hepatoprotective, anticarcinogenic, anti-inflammatory, antimicrobial, antiviral, vasodilatory, and antithrombotic effects of ferulic acid [69]. Vanillin (4-hydroxy-3-methoxybenzaldehyde) is a natural aromatic compound primarily used to impart fragrance and flavour to food products and beverages. In recent years, scientific data have been compiled on vanillin’s anticancer, antidiabetic, antioxidant, anti-inflammatory, and antimicrobial effects [70]. Vanillin is one of the components of propolis, contributing not only to a pleasant aroma but also to its biological impact. The smallest quantities of vanillin and cinnamic acids have been detected in the studied extracts. Vanillic acid and cinnamic acid are phenolic acids, which are secondary aromatic products known for their strong antioxidant activity [71].

Our research group found that the investigated propolis extracts exhibited excellent antiradical activity. Overall, our research data indicated that antiradical activity directly correlates with the concentrations of active compounds in the extract. We used a simple and quickly performed method to evaluate antioxidant activity. Our chosen method confirmed the antioxidant activity of the extracts. The DPPH method is suitable for identifying antioxidants that are soluble in organic solvents. The DPPH radical is sensitive to light, oxygen, and pH changes, leading to varying results when different solvents are used [72]. According to the data obtained during this study, the examined extracts were effective antioxidants in vitro.

The results confirm published research data indicating that Baltic propolis extracts exhibit high antioxidant activity attributable to their chemical composition [73,74]. Our research findings demonstrated that the antioxidant activity of propolis extracts directly depended on the quantity of active compounds present. A strong correlation was established between the number of active compounds and antioxidant activity.

After examining the scientific literature, we can conclude that antimicrobial activity is one of propolis’s most studied biological effects [17,30,64,65,66,75,76,77,78]. This is particularly relevant given the high resistance of some microorganisms to antibiotics and antifungal drugs, posing a global threat [79,80,81]. Betancourt and other researchers made a remark that propolis can be successfully used to treat eye diseases in cats and dogs; e.g., propolis drops have been used for the treatment of eye diseases such as conjunctivitis, corneal ulcers, and keratoconjunctivitis, for up to seven days in acute cases and up to ten days in chronic cases [82]. Cardoso and other scientists also used propolis as ear drops when *S. aureus* was isolated from dogs with otitis [83]. The results of these studies confirmed the antimicrobial activity of propolis. They provided additional information on the biological properties of propolis extracts and their potential use in the fight against antimicrobial resistance (AMR) [84,85]. Various studies on the antibacterial efficacy of ethanolic propolis extracts correlate with the active compounds found in the extracts [86].

After conducting an in vitro antimicrobial evaluation study with ethanolic propolis extracts, we observed that the ethanolic propolis extracts did not inhibit *Enterococcaceae*, *Enterobacteriaceae*, and *Pseudomonadaceae* family bacteria. The results of our study showed that the propolis ethanol extracts (Lithuania, Latvia, Poland) are effective against *S. auresus*, *S. agalactiae*, *B. cereus.* The diameters of the sterile zones of the clinical strains were 13.33 ± 1.15–26.33 ± 1.15 mm, and those of the reference were 14 ± 1.15–22 ± 2 mm. The researchers believe that propolis has weaker activity against Gram-negative bacteria due to its multi-layered structure and higher fat content in the cell wall [87,88]. Polish researchers reported that propolis showed activity against various bacterial strains, including Gram-positive bacteria (*S. aureus*, *Staphylococcus* spp., *Bacillus subtilis*, and *Enterococcus faecalis*) and Gram-negative *Enterobacter colica* (*E. coli*, *P. aeruginosa*) [25,26,27]. However, upon analyzing Nepali propolis, scientists noticed it exhibited similar antibacterial activity against Gram-negative and Gram-positive bacteria [32]. Propolis from the Middle East was also found to be highly active against both Gram-positive (*Staphylococcus aureus*) and Gram-negative (*Escherichia coli*) strains [30]. In general, there is a lack of studies investigating the antimicrobial activity of non-ethanolic extracts. The examined extracts, made from water and poloxamer (P407), did not inhibit the growth of *Staphylococcaceae*, *Streptococcaceae*, *Bacillaceae*, *Enterococcaceae*, *Enterobacteriaceae*, *Pseudomonadaceae*, and *Saccharomycetaceae* family isolates. This could be attributed to the low levels of active compounds isolated. The mixture of poloxamer and water was not an effective solvent for extracting the active components of propolis compared to other solvents we tested and to the aqueous extract of propolis with PEG additive evaluated by other researchers [78]. However, the Lithuanian aqueous propolis extract with a PEG additive exhibited antibacterial effects. Non-ethanolic extracts could serve as an excellent alternative to ethanolic ones for propolis extracts, provided that the solvent chosen for their production effectively separates the active compounds from the raw material. This was confirmed by experimental studies conducted by our research team using a DES solvents to produce propolis extracts. Although DESs are increasingly used for extracting active compounds from plant and animal raw materials, it should be noted that the extraction of propolis active compounds with these solvents is limited. Greek researchers studied the effect of different DESs on the yield of active compounds and antioxidant activity but did not investigate the antimicrobial activity of the extracts [89]. Our results show that the eutectic extracts of propolis DES were effective in inhibiting all Gram-positive and Gram-negative bacterial strains tested. The results of our study showed that propolis DES extracts (Lithuania, Latvia, Poland) effectively inhibited the growth of clinical and reference *S. aureus*, *S. agalactiae*, *B. cereus*, *Enterococcus feacalis*, *E. coli*, and *P. aeruginosa*. The diameters of the zones of inhibition in the clinical strains were 30.33 ± 1.52–40.33 ± 1.52 mm, and those of the reference were 31.33 ± 1.52–40.66 ± 1.52 mm. Radošević et al. investigated the antibacterial activity of DESs solvents with choline chloride and found that this solvent effectively inhibited the growth of *S. aureus* [38]. Bedair et al. also found that a DES with choline chloride has potential antimicrobial activity against *Enterococcus feacalis* strains [90]. According to the researchers, Gram-negative bacteria are less sensitive because of their outer membrane in the cell wall. This may be the reason why eutectic solvents were less inhibitory to *E. coli*, *P. mirabilis*, *S. typhimurium*, and *P. aeruginosa* than *S. aureus* [38].

A review of the scientific literature suggests that the MIC values of propolis can vary due to differences in geographical origin and composition across various countries [91,92,93,94,95,96,97]. The antibacterial efficacy of selected propolis extracts (ethanol and a DES) and *p*-coumaric acid at minimum inhibitory concentration (MIC) was determined against *Staphylococcus aureus*, *Streptococcus agalactiae*, *Bacillus cereus*, *Enterococcus faecalis*, *Escherichia coli*, and *Pseudomonas aeruginosa*, as well as clinical and reference bacterial strains. The MIC values of the propolis DES extracts were lower than those of the conventional ethanolic propolis extract. In our study, the minimum inhibitory concentration of the propolis DES extract was lower against Gram-positive bacterial strains (50–500 µg/mL). Higher concentrations of the propolis DES extract were required for Gram-negative bacterial strains, with 1000 µg/mL readings. The differences in antibacterial efficacy against Gram-positive and Gram-negative bacteria reported in the literature [96,98,99] corroborate our findings, with the MIC values for Gram-positive bacteria ranging from 3.125 to 400 µg/mL. Comparison of our data with those of Trusheva et al., who also studied the antimicrobial activity of NADES solvents, showed similar MIC values against *S. aureus* [100]. The MIC values of the ethanolic propolis extract were also found to be lower against Gram-positive bacterial strains (500–1000 µg/mL) compared to Gram-negative bacterial strains (5000 µg/mL). Polish researchers conducted a study on the antimicrobial activity of ethanolic propolis extract (EEP). They reported the highest activity against *S. aureus* in EEP from Turkey, Taiwan, and Oman, with the MIC values of 8, 10, and 81 µg/mL, respectively, and the lowest activity in propolis samples from Chile, Australia, and Germany, with the MIC values of 1445, 1200, and 750 µg/mL, respectively. Propolis extracts in ethanol from Turkey, Oman, and Slovakia were the most active against *E. coli*, with the MICs of 116, 302, and 510 µg/mL, respectively. Propolis samples from Germany, Korea, and Ireland had the lowest activity, with the MICs of 1200–5000 µg/mL [30]. Romanian and Brazilian researchers have also found that propolis ethanol extracts are considered viable synthetic products for treating canine superficial dermatitis and otitis caused by staphylococci [97,101]. However, a study by Grecka et al. revealed significant differences in the activity of ethanolic extracts of Polish propolis against Gram-positive and Gram-negative bacteria. Up to a 4096 µg/mL concentration, the researchers observed no activity against the *E. coli* and *P. aeruginosa* strains studied [92]. In our study, the MIC of *E. coli* and *P. aeruginosa* was 5000 μg/mL. Al-Ani et al. investigated European propolis samples from different geographical origins, finding both antimicrobial properties (the MICs against Gram-positive microorganisms ranged from 0.08 mg/mL to 2.5 mg/mL) and similarity to the minimum inhibitory concentration (0.5–1 mg/mL) of the ethanolic propolis extract studied in our group [102]. The results with *p*-coumaric acid demonstrated antimicrobial activity as a single active compound, with the readings ranging from 100 to 1000 µg/mL. Comparing these results with the MIC of Brazilian propolis prenylated *p*-coumaric acid, similar efficacy was observed against Gram-positive bacterial strains (*S. aureus* 100–300 µg/mL) [103]. A group of US researchers found, similar to our study, that *p*-coumaric acid had inhibitory effects on *E. coli* at 1000 µg/mL, *S. aureus* at 500 µg/mL, and *B. cereus* at 500 µg/mL [104]. The primary reason for the differing results compared to the literature is propolis’s geographically variable chemical composition.

To assess the efficacy of propolis extracts, we evaluated their antifungal activity using the agar diffusion method against *C. albicans* [61,78]. The results of our study showed that both ethanolic and DES propolis extracts (Lithuania, Latvia, Poland) had antifungal activity. The diameters of the inhibitory zones of the ethanolic propolis extracts have been measured at 12.66 ± 1.52 to 17 ± 1.73 mm, and those of the DES propolis extracts have been measured at 21 ± 1 to 33.66 ± 1.52 mm. Turkish scientists reported that propolis also inhibits the growth of some clinical strains of bacteria and yeasts [105]. Fuentes Esquivel and other researchers investigating the antifungal activity of Mexican propolis in canine otitis showed that propolis extracts exhibited antifungal effects [106]. A study was also conducted in which dogs with dermatophytosis were treated with propolis-based soap at weekly bathing intervals of three to eight baths. After the study, it was observed that after two weeks of treatment, the dogs recovered from the lesions [107].

This study’s results confirmed that the eutectic solvent components possess antimicrobial properties. Portuguese scientists examined extracts prepared based on NADES, which included lactic acid in their composition. During the study, they discovered that lactic acid introduced into the composition of eutectic solvents exhibits antimicrobial activity against Gram-negative and Gram-positive bacterial strains [108]. Furthermore, Greek scholars attempted to compare the antimicrobial efficacy of natural organic acid and found that lactic acid (LA) also exhibits antimicrobial efficacy [109]. The latest research data from scientists showed that the use of DES extracts with choline chloride and lactic acid not only resulted in the highest extraction efficiency but also demonstrated antimicrobial efficacy against *Salmonella*, *S. aureus*, *E. coli*, *P. aeruginosa*, and *B. subtilis* [41]. A eutectic solvent with choline chloride and lactic acid emerges as a potential carrier of active compounds in producing non-ethanol propolis extracts.

## 5. Conclusions

The findings of this study indicate that the isolation of phenolic compounds is feasible in producing propolis extracts using a eutectic solvent. A eutectic solvent based on choline chloride and lactic acid emerges as a potential carrier of propolis active substances, capable of enhancing the antibacterial effect of propolis active compounds. The antioxidant activity of propolis extracts directly depends on the extracted amounts of active substances. Propolis extracts from the Baltic region exhibit a similar chemical composition and demonstrate consistent biological effects. Our study confirms that *p*-coumaric acid dominates the chemical composition of propolis extracts from the Baltic region. Based on the results of this study regarding the antibacterial and antioxidant activities, further development in this area holds promise for integrating and expanding the practical application of ethanolic and DES propolis extracts in veterinary medicine, particularly for treating antibacterial and anti-inflammatory diseases.

## Figures and Tables

**Table 1:** Propolis HPLC analysis of extracts.

Solvent	Propolis	Vanillic Acid (mg/g)	Vanillin (mg/g)	Ferulic Acid (mg/g)	Cinnamon Acid (mg/g)	*p*-Coumaric Acid (mg/g)
Ethanol	1 LT	28.63	587.49	693.18	36.05	1506.65
	SD	2.37	33.86	27.83	3.98	91.39
	2 PL	23.16	463.10	473.59	21.12	1155.90
	SD	0.20	2.99	15.02	0.99	21.57
	3 LV	22.23	574.51	593.26	38.83	1415.98
	SD	0.94	19.62	22.82	0.23	73.92
Eutectic	4 LT	19.61	404.78	315.54	16.29	954.76
	SD	0.40	13.49	8.30	0.01	51.95
	5 LV	3.19	141.03	112.79	7.41	321.13
	SD	0.19	0.42	1.62	0.28	10.44
	6 PL	5.28	168.84	89.06	5.18	328.05
	SD	0.27	3.46	5.24	0.09	17.33
P407	7 LT	5.30	-	6.05	0.49	14.94
	SD	0.22	-	0.05	0.00	0.03
	8 PL	7.26	-	1.48	-	5.34
	SD	0.40	-	0.08	-	0.37
	9 LV	0.53	14.36	10.93	0.87	30.80
	SD	0.01	1.33	0.12	0.01	1.79

**Table 2:** Minimum suppressive concentrations of propolis extracts against the bacterial strains tested.

	Eutectic Propolis Extract		Ethanol Propolis Extract		*p*-Coumaric Acid	
	MIC Reference Strains (µg/mL)	MIC Wild Strains (µg/mL)	MIC Reference Strains (µg/mL)	MIC Wild Strains (µg/mL)	MIC Reference Strains (µg/mL)	MIC Wild Strains (µg/mL)
*S. aureus*	50	100	500	1000	200	500
*S. agalactiae*	200	200	1000	500	100	200
*B. cereus*	120	200	1000	500	500	500
*E. feacalis*	500	500	1000	1000	200	500
*E.coli*	1000	1000	5000	5000	500	1000
*P. aeruginosa*	1000	1000	5000	5000	500	1000

## Data Availability

The data presented in this study are openly available in the article.
